# Supplementary material for: bin3C: exploiting Hi-C sequencing data to accurately resolve metagenome-assembled genomes
Source: Genome Biol. 2019 Feb 26;20:46. doi: 10.1186/s13059-019-1643-1 (PMC6391755; doi:10.1186/s13059-019-1643-1)
Supplement: Supplementary file 2 — Supplementary figures and tables. Additional figures S1-S4 and tables S1-S2 supporting the manuscript. (PDF 2836 kb) [file 13059_2019_1643_MOESM2_ESM.pdf]

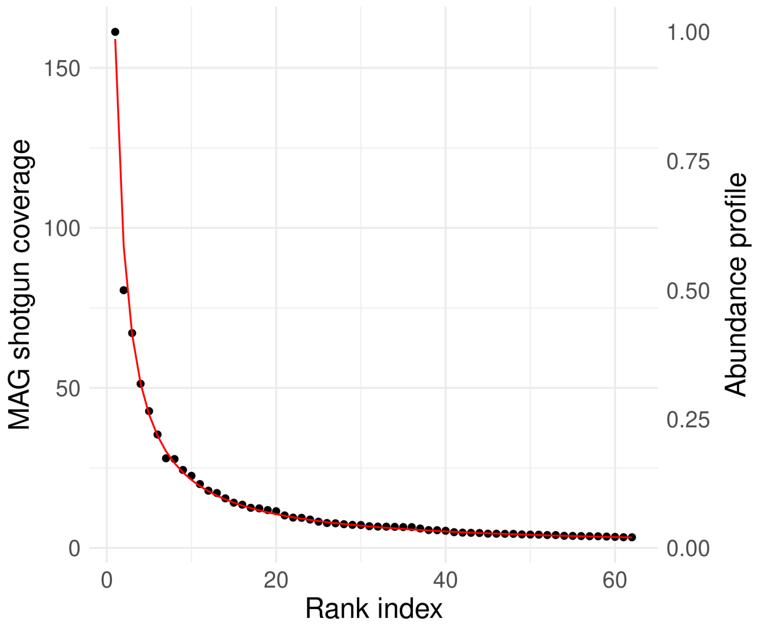

Figure S1: Relative abundance of the simulated community was modelled as a Generalized Pareto distribution (red curve). After genome binning was completed, the estimated coverage of MAGs (black circles) agrees closely with the input abundances. Here we have defined the most abundant member as equal to unity.

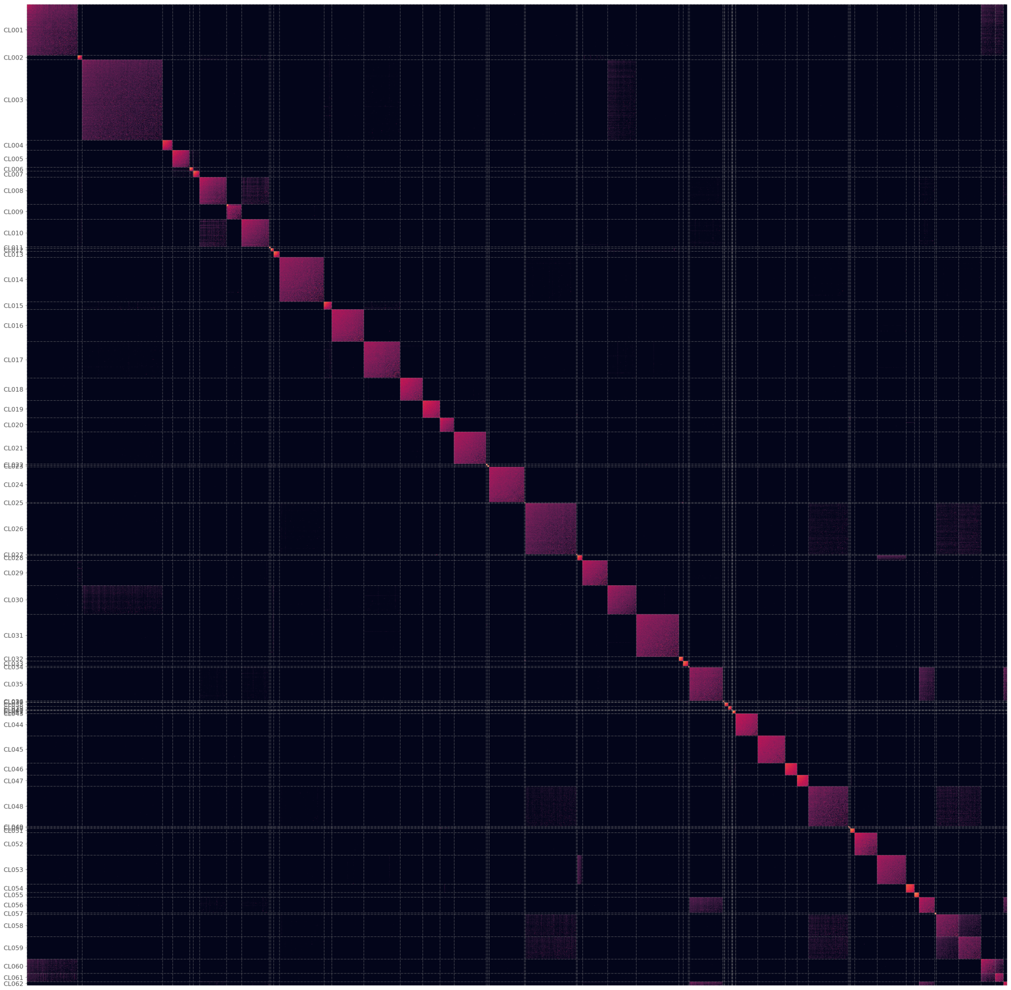

Figure S2: The contact map of the simulated community at 250x shotgun and 200M Hi-C pairs. Here, intensity of a pixel is equal to the natural log of the normalized interaction strength between two contigs. When clustered, the the heatmap appears in block diagonal form, where each block represents a cluster. Each cluster is sorted largest to smallest contig, giving the impression of a gradient which is only an artefact. Blocks are proportional to the number of contigs.

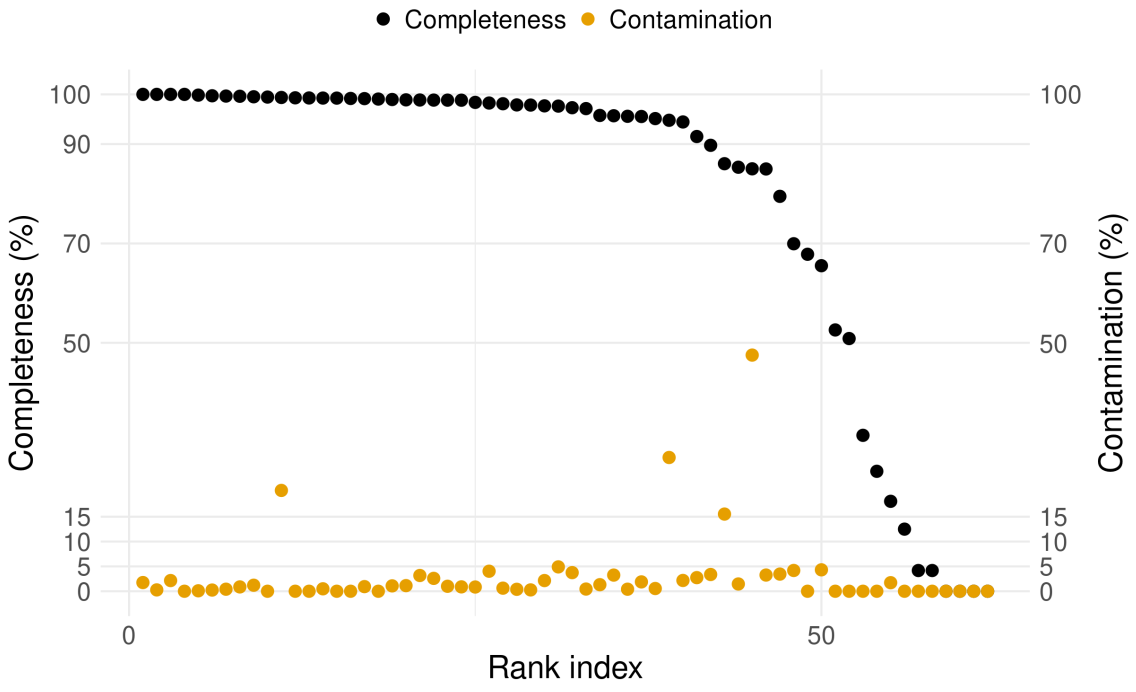

Figure S3: Completeness and contamination plot of the full depth 250x/200M pair run of the simulated community. There were 62 clusters in the solution from an initial 63 genomes. Ticks along the y-axis mark thresholds used in the simple CheckM standard for MAG quality. Completeness (>90, >70, >50) and Contamination (>5, >10, >15).

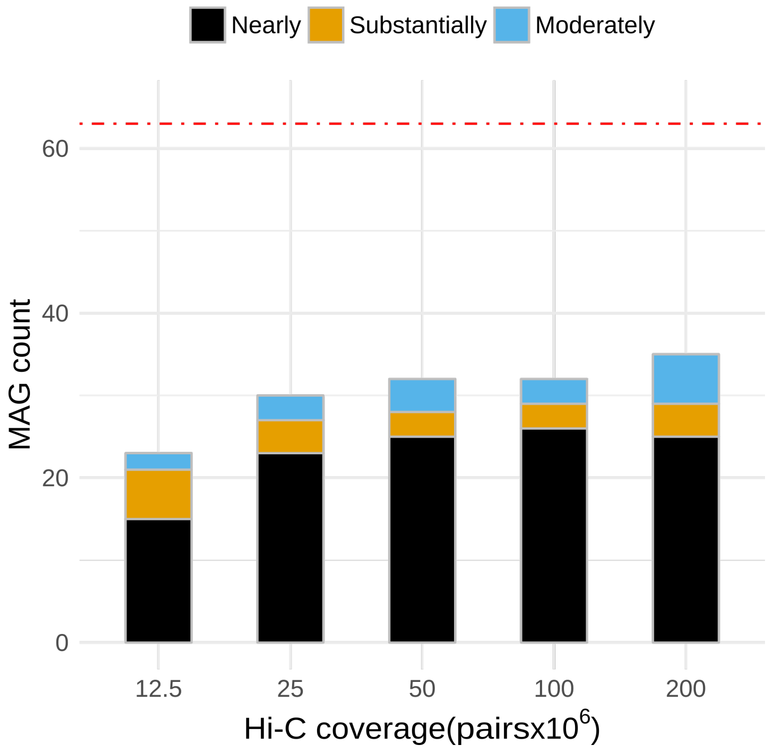

Figure S4: MAGs retrieved from the simulated community when shotgun coverage is reduced by half (125x most abundant genome). At the maximum Hi-C depth of coverage, CheckM estimated that there were 25 nearly, 4 substantially and 6 moderately complete genomes.

	Simple MAG Rank								
	Nearly			Substantially			Moderately		
**Statistic**	**min**	**max**	**median**	**min**	**max**	**median**	**min**	**max**	**median**
Contigs	14	294	83	35	495	109	41	416	151
Coverage	5.9	447.5	34.4	4.3	416.4	22.1	3.7	83.4	21.6
N50	13,103	297,079	73,278	6,203	169,501	38,287	5,009	74,774	17,627
Extent	1,681,638	4,967,006	2,810,566	1,555,274	5,460,325	2,480,584	1,224,207	3,402,418	1,836,393
gc_expect	34.40	62.82	52.88	28.18	64.99	44.54	35.92	60.07	49.60

Table S4: Summary statistics for MAGs retrieved using bin3C from a real human gut microbiome, divided into ranks as defined by Parks et al based only on completeness and contamination

	GSC MIMAG Rank					
	High quality			Medium quality		
**Statistic**	**min**	**max**	**median**	**min**	**max**	**median**
Contigs	27	275	85	14	495	92
Coverage	10.7	447.5	68.38	3.7	416.4	25.6
N50	31,316	221,523	75159	5,009	297,079	52,246
Extent	1,863,635	4,099,346	2549586	1,224,207	5,460,325	2,623,866

Table S5: Summary statistics for MAGs retrieved using bin3C from a real human gut microbiome, divided into ranks defined by the GSC MIMAG standard.
