# Supplementary material for: bin3C: exploiting Hi-C sequencing data to accurately resolve metagenome-assembled genomes
Source: Genome Biol. 2019 Feb 26;20:46. doi: 10.1186/s13059-019-1643-1 (PMC6391755; doi:10.1186/s13059-019-1643-1)
Supplement: Supplementary file 5 — Reviewer reports. (DOCX 87 kB) [file 13059_2019_1643_MOESM5_ESM.docx]

**First round of review**

**Reviewer 1**

**Are the methods appropriate to the aims of the study, are they well described, and are necessary controls included?**
Yes

**Are the conclusions adequately supported by the data shown?**

Yes

**Are sufficient details provided to allow replication and comparison with related analyses that may have been performed?**

Yes

**Does the method perform better than existing methods (as demonstrated by direct comparison with available methods)?**

Yes. The new method is fairly benchmarked against the nearest competitor and appears superior.

**Is the method likely to be of broad utility? Is any software component easy to install and use?**

Yes. Clean repository and documentation. The nearest competing software is proprietary, and so this open alternative is readily welcomed.

**Is the paper of broad interest to others in the field, or of outstanding interest to a broad audience of biologists?**

Yes: Hi-C metagenome binning is a state-of-the-art technique that will be of great interest to many readers.

**Comments to authors**

This is a beautifully written paper that is incredibly clear and easy to follow. The experiments are excellent and performed on both simulated and real data using standard measurements. The methods are well-thought-out and demonstrate the authors' expertise. The software is openly available and documented (as well as all of the data from the paper with assigned DOIs). The discussion provides a balanced review of limitations and helpful recommendations. In summary, I can find very little to criticize. A few trivial comments follow:

- Page 8: It would be helpful to add reference for B-Cubed when it is first mentioned (Bagga and Baldwin, 1998).

- Page 18: This sentence does not parse "A wide variation such aspects as in the size of clusters and weight of intra-cluster edges relative to the whole graph make a complete reconstruction difficult for algorithms with limited resolution."

- Figure 7 caption says "When compared by ranking standards, based either on measures of completeness and contamination only" but the figures appear to only address completeness and not contamination.

**Reviewer 2**

**Are the methods appropriate to the aims of the study, are they well described, and are necessary controls included?**
Yes

**Are the conclusions adequately supported by the data shown?**

Yes

**Are sufficient details provided to allow replication and comparison with related analyses that may have been performed?**

Yes

**Does the method perform better than existing methods (as demonstrated by direct comparison with available methods)?**

The authors compare to the only available software for binning on Hi-C data, and it does perform significantly better. In addition, the software presented here is, in contrast to the previously existing one, open source.

**Is the method likely to be of broad utility? Is any software component easy to install and use?**

Likely broad utility. I haven’t had time to try to install or run it though.

**Is the paper of broad interest to others in the field, or of outstanding interest to a broad audience of biologists?**

Yes: Broad interest to others in the field.

**Comments to authors**

I much enjoyed reviewing this manuscript since it is very well written, the results are presented in a logical and clear manner, and the conclusions are sound.

Consequently, I only have minor comments/suggestions:

1. Figure 2 shows that recall increases substantially with Hi-C coverage, while the increase in the number of retrieved nearly complete MAGs in Figure 3 is more moderate. This discrepancy is explained by that the number of contigs in the nearly complete MAGs increase from n=94 to n=179 going from lowest to highest coverage. This could indicate that the marker gene based classification is very imprecise. However, only writing out the number of contigs here may be misleading and I suggest that you also write the number of base pairs. My guess is that the additional contigs gained at higher coverage are generally smaller than the ones gained at lower coverage, hence the completeness of the MAGs may not increase as much as the number of contigs may indicate. So what then could explain the discrepancy between the two figures? Maybe it is a matter of how recall is defined. This is not specified in detail in the manuscript - but I think it should be, as well as should the other metrics used - but I assume it is calculated by for each ground truth genome, finding the bin where the greatest number of contigs (n) for this genome is included. Then the n:s for all genomes are summed up and this sum is divided by the total number of contigs in the dataset. In the case of low Hi-C coverage there are probably a large number of very incomplete bins consisting of primarily small contigs that contribute to the low recall. If you instead weight the recall values by the sizes of the contigs, these small contigs will have a lower influence on the recall values. This may give better correspondence between the two graphs and I think it is more relevant to judge the success of the binning on a per base premise rather than on a per contig premise.

2. Traditional metagenomic binning can be improved by splitting long contigs into smaller pieces. By doing so, the data within a genome behaves more uniformly, which can improve the outcome of the clustering. One will also get more data points per cluster, which should likewise be beneficial for the clustering. In the CONCOCT algorithm for example, contigs >= 20,000 bp are split into 10,000+ pieces (hence a contig of 39,000 bp is split into 10,000, 10,000 and 19,000 bp long contigs). After the clustering, a majority vote can be used to decide the affiliation of a contig, in case the contig is distributed over multiple clusters. I think this approach would be worth testing also in your case when clustering the contact map.

3. p.5 l.9. Please cite the article where the term MAG was first introduced (Hugerth et al. Genome Biol. 2015)

4. p.12 l.58. Should it be "tends to decrease with increasing problem size"? If you randomly group equally sized contigs of two equally sized genomes into two bins, half of the contigs will be correctly binned. Doing the same on four genomes into four bins will give ¼ of the contigs correctly binned.

5. p.14 l.55. Should it be "real read-sets"?

6. p.18 l.34-35. Unclear to me what "communities" refer to here. If it is the pangenome of a species, "population" may be a better word. "Community" should refer to the whole collection of pangenomes in a sample.

7. Figure 3. Switch order in color legend to "Nearly", "Substantially", "Moderately" (as in Figure 7A)

**Response to reviewers**

**Reviewer #1**: This is a beautifully written paper that is incredibly clear and easy to follow. The experiments are excellent and performed on both simulated and real data using standard measurements. The methods are well-thought-out and demonstrate the authors' expertise. The software is openly available and documented (as well as all of the data from the paper with assigned DOIs). The discussion provides a balanced review of limitations and helpful recommendations. In summary, I can find very little to criticize. A few trivial comments follow:

- Page 8: It would be helpful to add a reference for B-Cubed when it is first mentioned (Bagga and Baldwin, 1998).

Added, thanks.

- Page 18: This sentence does not parse "A wide variation such aspects as in the size of clusters and weight of intra-cluster edges relative to the whole graph make a complete reconstruction difficult for algorithms with limited resolution."

The sentence has been made more clear.

*Wide variance in structural features, such as the size of clusters and the weight of intra-cluster edges relative to the whole graph, make a complete reconstruction difficult for algorithms with limited resolution.*

- Figure 7 caption says "When compared by ranking standards, based either on measures of completeness and contamination only" but the figures appear to only address completeness and not contamination.

For simplicity, we are using equivalent completeness and contamination criteria when counting MAGs,

that is to say: 90/5, 70/10, 50/15. This has now been explained under Performance Metrics and in the table 1 legend.

**Reviewer #2**: I much enjoyed reviewing this manuscript since it is very well written, the results are presented in a logical and clear manner, and the conclusions are sound. Consequently, I only have minor comments/suggestions:

1. Figure 2 shows that recall increases substantially with Hi-C coverage, while the increase in the number of retrieved nearly complete MAGs in Figure 3 is more moderate. This discrepancy is explained by that the number of contigs in the nearly complete MAGs increase from n=94 to n=179 going from lowest to highest coverage. This could indicate that the marker gene-based classification is very imprecise. However, only writing out the number of contigs here may be misleading and I suggest that you also write the number of base pairs. My guess is that the additional contigs gained at higher coverage are generally smaller than the ones gained at lower coverage, hence the completeness of the MAGs may not increase as much as the number of contigs may indicate.

The large possible number of summary statistics which could be given were, we felt, a threat to the readability of the manuscript. We opted to use a small number of maximally informative summary statistics to make our points clear.

That said, as the number of Hi-C pairs increases, there was a steady rise in the total number of contigs in all genome bins: 2550 @ 12.5M Hi-C pairs to 6968 @ 200M (a 2.7x change). At the same time, the increase in total extent was relatively smaller: 122.4Mbp @ 12.5M to 145.7M @ 200M (a 1.2x change). Considering these statistics together (extent per contig), we see a decrease from 48.0 kbp/contig @ 12.5M to 20.9 kbp/contig @ 200M.

That the contigs gained with increased Hi-C coverage tend to be small is correct and these appear to go unnoticed by marker-gene measures of genome bin quality. It may be a surprising finding for some practitioners in our field that the CheckM metrics, which have become a *de facto* standard, have this potential shortcoming and it highlights a topic that would be interesting to explore more deeply in a future manuscript.

The manuscript sentence in question has been amended to be:

*To explain this, we referred to the cluster reports provided by bin3C, where for nearly complete MAGs, we found that the average number of contigs increased from 77 at 12.5M pairs to 179 at 200M pairs while the total number of contigs increased from 2550 to 6968.*

**Erratum please note:** when revising this section we discovered an copying error in our figures. The average number of contigs in nearly complete bins at 12.5M pairs was originally stated to be 94 but was in fact is 77. We could find no other such errors.

So what then could explain the discrepancy between the two figures? Maybe it is a matter of how recall is defined. This is not specified in detail in the manuscript - but I think it should be, as well as should the other metrics used - but I assume it is calculated by for each ground truth genome, finding the bin where the greatest number of contigs (n) for this genome is included. Then the n:s for all genomes are summed up and this sum is divided by the total number of contigs in the dataset. In the case of low Hi-C coverage, there are probably a large number of very incomplete bins consisting of primarily small contigs that contribute to the low recall. If you instead weight the recall values by the sizes of the contigs, these small

contigs will have a lower influence on the recall values. This may give better correspondence between the two graphs and I think it is more relevant to judge the success of the binning on a per base premise rather than on a per contig premise.

We agree that scoring metrics which treat contigs of very different lengths uniformly are prone to these issues. Of the two formulations we used, AMI ignores contig length information. Alternatively, weighted B-cubed does make use of contig length in calculating scores. We have revised the manuscript to highlight this deficiency in AMI (see below in green).

*While AMI treats all objects under study as having equal value, weighted B 3 allows the value of objects to vary, and we chose to use contig length as a representation of value for the problem of genome binning. Therefore, the weighted B 3 results measure the fraction of the genome binned accurately, not just the number of contigs.*

Returning to the reviewer comments immediately above, although those contigs which require higher coverage to be clustered correctly tend to be shorter and therefore of less value, external measures consider the totality of the clustering solution against the ground truth. A common mode of behaviour in clustering algorithms is to produce many small clusters when signal is insufficient. Even when these small clusters are of high homogeneity (high Precision), they will reduce the overall result (F-score) as they are far from complete representations (low Recall).

2. Traditional metagenomic binning can be improved by splitting long contigs into smaller pieces. By doing so, the data within a genome behaves more uniformly, which can improve the outcome of the clustering. One will also get more data points per cluster, which should likewise be beneficial for the clustering. In the CONCOCT algorithm for example, contigs >= 20,000 bp are split into 10,000+ pieces (hence a contig of 39,000 bp is split into 10,000, 10,000 and 19,000 bp long contigs). After the clustering, a majority vote can be used to decide the affiliation of a contig, in case the contig is distributed over multiple clusters. I think this approach would be worth testing also in your case when clustering the contact map.

We appreciate the suggestion and have spent time evaluating the effects of the proposed contig splitting procedure. Although our findings so far have shown mixed outcomes depending on how the resulting solutions are evaluated, they provide motivation for future work.

In more detail using the simulated community:

Assessing the changes with CheckM after splitting longer contigs into chunks of 10 kbp and subsequently applying bin3C, we saw a slight improvement in terms of the number of “nearly complete” MAGs (>90%,<5%) (Table 1). However, at 5 kbp the number of “nearly complete” MAGs decreased to below that of the original unsplit analysis. Although all three tests produced between 40 to 42 bins with >= 90% completeness, the 5 kbp test suffered from an increased number of bins with > 5% contamination.

We then assessed solutions using the external metrics (B-cubed, etc). We found that splitting at 10 and 5 kbp led to an increasing improvement on Recall and Precision (Table 1), with the 5 kbp test possessing the best Precision, Recall and F-score.

**Table 1.** Changes in B 3 and CheckM for different chunking size on the simulated community.

We assessed where these improvements were occurring and found that this was in part due to the presence of “misassembled” contigs that contain sequence from different mixtures of source genomes. The impact is complicated by the fact that the set of contigs not only forms the basis of bin3C genome binning, they are also the basis of the ground truth inference.

Although our ground truth assignment supports overlapping clusters, the objects under consideration (contigs or pieces thereof) are treated wholly and therefore the process of attributing source genome(s) is carried out over their full extent.

For co-assembled contigs (those which are a composite of multiple source genomes) the model cannot accommodate the existence of discontinuous changes in the set of contributing source genomes over small but not insignificant regions. Though it is community composition dependent, with communities containing high levels of strain heterogeneity likely to be more error-prone, this limitation in how ownership must be modelled in the ground truth is responsible for systematic defects.

By chopping contigs into smaller pieces, both the Hi-C mediated clustering and sequence alignment based ground truth inference are granted increased freedom in their independent fitting processes. Together, both appear to be responsible for the improvement seen in external measures on simulated data.

As it stands, a simplistic chopping process does not make use of the evidence available within a genome sequencing project. Consequently, it will introduce errors in the form of contamination when the coordinates of chopping points to not correspond to the true points of transition in genomic source (e.g. the points of misassembly or structural variation among strains). Rather, we envision that this could be significantly improved upon by an approach which first attempts to determine these points of change. This goes to emphasise where we would like to focus our attention in the near future, either by inferring the true change points via analysis of mapped reads or avoiding the need for this by working directly with the assembly graph rather than its linearized representation as contigs.

As a result of this work, we have amended the comments in limitations as follows.

First, for the ground truth:

*The ground truth as determined in our work is imperfect and this has an impact on the extrinsic validation metrics. Notably, when a simulated community possesses multiple strains of a single species, parts of a contig can derive from one strain while other parts of the same contig may be a coassembly of multiple strains, and this is not accommodated by the ground truth.*

And the idea of splitting contigs, for which we have added a simple helper tool called split_ref.py to the bin3C code base:

*As assembly contigs are treated wholly in the genome binning process, errors due to misassembled contigs that combine sequence from different strain or species genome(s) are propagated into the bin3C output. To compensate for this, we have provided an optional helper tool (\texttt{split\_ref.py}) which can be used to uniformly split longer contigs into smaller pieces of a target length. Using this approach on our simulated community with target lengths of 10 kbp and 5 kbp, we saw a marked improvement in $\mathrm{B}^3$ Precision and Recall. We simultaneously observed a detrimental increase in MAG contamination inferred by CheckM when these fragments became small (5 kbp) and little change in MAG completeness, suggesting that deeper investigation may be warranted. In future work, one might take better advantage of the shotgun sequence data to identify potential locations of strain- or species- misassembly and coassembly and to target contig splitting, or alternatively, apply \mbox{Hi-C} reads to the assembly graph to deconvolve the graph itself.*

3. p.5 l .9. Please cite the article where the term MAG was first introduced (Hugerth et al. Genome Biol. 2015)

Added, thanks

4. p.12 l.58. Should it be "tends to decrease with increasing problem size"? If you randomly group equally sized contigs of two equally sized genomes into two bins, half of the contigs will be correctly binned. Doing the same on four genomes into four bins will give ¼ of the contigs correctly binned.

The statement has been reworded to be more clear

*AMI is a normalized variant of mutual information which corrects for the tendency of the number of random-chance agreements between clusters to increase as the number of clusters increases for a given problem size.*

5. p.14 l.55. Should it be "real read-sets"?

Corrected, thanks

6. p.18 l.34-35. Unclear to me what "communities" refer to here. If it is the pangenome of a species, "population" may be a better word. "Community" should refer to the whole collection of pangenomes in a sample.

Here we are using the term “community” as commonly used in the graph & clustering algorithms literature. That we were referring to the communities in the graph has been clarified in the text as follows (change underlined):

*This is because resolving all of the structural detail (all of the clusters) becomes an increasingly fine-grained task a graph grows in size and number of clusters.*

7. Figure 3. Switch order in color legend to "Nearly", "Substantially", "Moderately" (as in Figure 7A)

Fixed, thanks
